# *In vivo* evaluation of post-operative pain with different NiTi systems after root canal retreatment on single-rooted teeth

**DOI:** 10.6026/9732063002001106

**Published:** 2024-09-30

**Authors:** Swati Priya, Rana K.Varghese, Raunak Singh, Minakshi Sahu, Shreeya Jaiswal, Aditee Agrawal

**Affiliations:** 1 Department of Conservative Dentistry and Endodontic, New Horizon Dental College and Research Institute, Sakri, Bilaspur, Chhattisgarh, India

**Keywords:** Rotary, reciprocating, NiTi, retreatment, postoperative pain

## Abstract

The impact of different NiTi systems, such as ProTaper Gold, NeoEndo Flex, Hyflex EDM, Reciproc Blue and WaveOne Gold, on postoperative pain of single-rooted teeth following root canal retreatment is of interest to dentist. Additionally, the study aims to ascertain whether age, gender and tooth localization adhering to retreatment with various rotary and reciprocating instruments significantly affect postoperative pain. Root canal re-treatment with 5 (five) distinct rotary or reciprocating NiTi systems did not significantly affect postoperative pain or painkiller consumption. Postoperative pain levels in this trial peaked on the first day and then dramatically declined each subsequent day.

## Background:

The rapid advancements in endodontics present a "good news-bad news dilemma." The "good" news is that the collaboration of endodontics, periodontics and restorative dentistry saves millions of teeth annually. However, the "bad" news is that tens of millions of endodontically treated teeth fail each year due to various causes. Addressing these failures will be crucial for the future of endodontics. Globally, root canal therapy boasts a high success rate, preventing the extraction of billions of teeth. However, rare cases of failure occur, primarily due to inadequate cleaning and obturation. In such cases, non-surgical retreatment, which involves removing the original root canal filling to allow for additional cleaning and re-obturation, is the most conservative treatment option [1]. According to Ingle's "Washington Study", 60% of endodontic failures are due to incomplete root canal obturation, while 40% are attributed to root perforation, on-going trauma, fractured instruments, unfilled root canals, overfilled or overextended root canals, and other minor causes such as the accidental removal of silver points [2]. The American Association of Endodontics (AAE) defines endodontic retreatment as the process of removing the root canal filling from the tooth, followed by cleaning, shaping, and obturating the canals [3]. Inter-appointment complications following root canal retreatment (RCR) are more common than those following Initial Root Canal Treatment (IRCT). If the previous root canal filling is not thoroughly removed, residual infection may worsen due to imbalances in host-bacteria relationships or microbial interactions [4]. Accessing the root canal system for additional cleaning requires the removal of gutta-percha and sealer, which necessitates the elimination of infected dentin, necrotic tissue and bacteria [5]. The choice of the best file system is critical for efficient removal of obturation material from the root canal. Various methods, such as lasers, heat-bearing devices, nickel-titanium (NiTi) rotary tools, ultrasonic instruments and stainless steel hand files, are available for gutta-percha removal. Rotary devices, in particular, reduce clinical time during retreatment procedures [6]. Postoperative pain management is crucial for patient comfort, even though there is no direct correlation between postoperative pain and the long-term outcomes of root canal therapy [7]. Root canal retreatment aims to treat apical periodontitis after the failure of the original root canal therapy. The incidence of postoperative pain and flare-ups is significantly higher with root canal retreatment compared to initial root canal therapy. Factors influencing postoperative pain include age, gender, tooth type, pulpal and periradicular state, sinus tracts, history of postoperative pain, and the extent of chemical, mechanical, or bacterial damage during root canal preparation. While bacterial virulence and host-dependent immunologic factors are beyond the operator's control, technical parameters like chemo-mechanical disinfection methods and instruments can be managed by the operator [8]. Numerous technologies and methods are available for root canal retreatment, with engine-driven equipment providing practitioners the necessary tools for root canal contouring and root filling removal [9]. However, the process is fraught with potential issues such as instrument separation, ledging, perforation and apical ejection of debris and gutta-percha, all of which can negatively impact treatment outcomes [10]. The design variations of file systems, along with their micro-hardness, flexibility, and deformation capacity, influence the amount of extruded debris, which directly correlates with the degree of periapical inflammation. Instruments' cross-sectional area variations can affect postoperative pain by increasing levels of neuropeptides like SP and CGRP. Therefore, these factors should be considered when selecting instruments for mechanical root canal procedures [11].

## Materials and Methods:

The study was conducted in the Department of Conservative Dentistry and Endodontics at New Horizon Dental College and Research Institute, Sakri, Bilaspur, Chhattisgarh, India. It included 180 adult patients, aged 18 to 59 years, from both genders. Patients were either treated at the department, referred from our satellite dental center or attended dental camps hosted by our institution at Jan Swasthya Sahyog, Ganiyari and Bilaspur. Ethical approval was obtained and all participants provided written informed consent. The study excluded individuals younger than 18 years or those with severe systemic diseases, Ibuprofen allergies, acute apical abscesses and recent use of analgesics, anti-inflammatory drugs, or antibiotics within the past seven days, periodontal pockets greater than 4 millimeters, or large intraradicular posts. Participants were randomly assigned to five groups (n=36 per group), focusing on single-rooted teeth with a single canal and a post-treatment disease diagnosis. Teeth selected for the study had primary root canal fillings that were inadequate or leaky according to standard endodontic guidelines or were 2-4 mm short of the radiological apex and had a periapical index score of 4. Exclusions included teeth with open apices, intraradicular posts, sinus tracts, deep periodontal pockets (probing depth > 4 mm), systemic diseases (ASA III-VI), or a history of recent antibiotic or analgesic use. Root canal retreatment involved using 2% lignocaine with 1:100000 adrenaline for anesthesia. Coronal restorations and cavities were removed with sterile high-speed round burs under water cooling and rubber dam isolation. Canal filling materials were removed from the coronal third using Gates Glidden drills and working lengths were determined with a size 15 K-file, verified by a Root ZX Apex locator and dental X-ray. Root canal fillings were removed with an X smart plus endomotor and ProTaper Universal Retreatment files. Participants were allocated to five groups based on the final apical preparation instruments: ProTaper Gold (Group 1), HyFlex EDM (Group 2), NeoEndo Flex (Group 3), Reciproc Blue (Group 4) and WaveOne Gold (Group 5). No solvents were used during the retreatment, and apical patency was maintained with a size 10 K-file. Irrigation was performed using 2.5% sodium hypochlorite, followed by a final rinse with 2.5% sodium hypochlorite, 17% EDTA and 0.9% normal saline, using a side-vented needle. Canals were dried with absorbent points and filled with gutta-percha using the lateral condensation technique with an epoxy resin-based sealant. Access cavities were restored with GIC. Post-operative pain was assessed using an 11-level numerical rating scale at 24, 48 and 72 hours after retreatment. Patients were instructed to take 400 mg of ibuprofen every six hours if needed and to record their pain levels and analgesic consumption, which were collected during follow-up visits. Data were compiled in Microsoft Excel 365 and analyzed using SPSS version 23.0. Continuous variables were expressed as mean ± standard deviation and categorical variables as frequency and percentage. Statistical tests included the Kruskal-Wallis test for intergroup comparisons, the Friedman test for intragroup comparisons, and the Tukey post-hoc test. A p-value of <0.05 was considered statistically significant.

## Results:

Five groups-three rotary file systems (ProTaper Gold, Hyflex EDM, NeoEndo) and two reciprocating file systems (Reciproc blue, WaveOne Gold) as well as three time points-24, 48 and 72 hours after retreatment for 78 (seventy-eight) male and 102 (one hundred two) female patients were used in the analysis. The patients were divided into five groups at random, with 36 patients in each category. The mean and standard values are displayed for the continuous variables. Both frequency and percentage are displayed for the categorical variables (Table 1). The acquired data were compared within groups using the Friedman test and between groups using the Kruskal-Wallis test. The Kruskal-Wallis test, chi-square test and one-way ANOVA are used to examine the relationships between post-operative discomfort, tooth location, age and gender. For the comparison, the Tukey post-hoc test was also employed. Statistical significance is attained when the p-value is less than 0.05. Friedman test and One-way-ANOVA test were used for intra-group comparison in order to determine whether there is a significant difference in postoperative pain based on NRS score within each group. For all patients in each group, the p-value determined on the first, second, third and seventh day was less than 0.001, indicating a significant difference in postoperative pain between the patients in that group (Table 2).

ANOVA and the Kruskal-Wallis test were used to compare groups. The Kruskal-Wallis test yielded a p value of 0.74 on the first day, 0.91 on the second, 0.86 on the third and 0.96 on the seventh. On the first day, the ANOVA test yielded a p value of 0.82, on the second day, 0.73, on the third day, 0.69 and on the seventh day, 0.89. The p-value for both tests indicated that Groups 1, 2, 3, 4 and 5 did not significantly vary in their postoperative discomfort. The greatest amount of pain following treatment was noted in the first 24 hours and in each group, the discomfort significantly decreased in the next 48, 72, and then on seventh day. At any point in time, there was no discernible relationship between postoperative discomfort and gender, age, or tooth location (p <0.05). Women only reported noticeably more severe pain than men at the 48-hour mark. Friedman and ANOVA were used to determine the p value for the number of analgesics (Ibuprofen 400 mg) used by each group in the 24-, 48- and 72-hour periods. This allowed for intragroup comparison. The p-value of less than 0.001 indicated a statistically significant variation in the amount of analgesics consumed by the patients in each group. At all-time points, there was no significant connection (p > 0.05) seen between analgesic consumption and gender, age or tooth location. At every time point evaluated, there were noticeable variations in the amount of analgesics used by the various groups (p < 0.05) (Table 3).

## Discussion:

This prospective randomized clinical trial compared three rotary systems and two reciprocating systems for root canal retreatment, evaluating the frequency, severity, and duration of postoperative discomfort. The study found that postoperative pain was not significantly influenced by the type of motion, whether reciprocating (Reciproc Blue, WaveOne Gold) or rotary (ProTaper Universal Retreatment + ProTaper Gold, HyFlex EDM, NeoEndo Flex). These findings align with previous research that compared rotary and reciprocation systems in root canal retreatment [12-18]. Further, Eyüboglu and Özcan (2019), reported that the One Shape rotary system significantly reduced postoperative discomfort compared to other systems 18. Apical preparation and deliberate widening of the apical foramen have been shown to reduce microbial burden in cases of apical periodontitis, leading to more favorable outcomes and less postoperative discomfort. Nonetheless, the apical extrusion of debris, including dentine chips, necrotic pulp tissue, bacteria and their by-products, can cause postoperative discomfort or long-term post-treatment complications. The severity of this inflammation depends on the amount of extruded debris and the pathogenicity of the bacteria [19]. Laboratory research comparing reciprocating and rotary systems during root canal retreatment has shown conflicting results regarding debris extrusion [20, 21]. Differences between the rotary and reciprocating systems may stem from variations in motion kinematics and the number of instruments used [19]. Neuropeptides SP and CGRP were found in higher concentrations in NiTi systems with a triangular cross-sectional design compared to those with an S-shaped cross-section, potentially linking debris extrusion with periodontal ligament inflammation during root canal therapy [22, 23]. Despite these considerations, this study found no significant difference in postoperative discomfort between the reciprocating and rotary systems. Several factors could contribute to this result, including the potential reduction of extrusion by the backpressure of periodontal tissues in clinical settings. Additionally, the original root canal fillings in this study were either under-obturated according to endodontic standards or positioned 2-4 mm short of the apical foramen, compared to 1-mm short in laboratory experiments. The closer proximity of the filling to the apical foramen could impact the extent of extruded debris. Other factors, such as bacterial pathogenicity and host-dependent inflammatory responses, may also influence postoperative pain [19].

## Conclusion:

The study evaluated postoperative discomfort and the effectiveness of reciprocating and continuous rotary NiTi devices during root canal retreatment. Both kinematic motions demonstrated comparable performance in root canal debridement. However, the reciprocating system, which utilizes a single NiTi file with enhanced flexibility and reduced fatigue, completed the procedure more quickly, resulting in a 62% reduction in preparation time. Despite differences in design and usage, no significant difference in postoperative pain was observed between the systems. Other factors, such as instrument design and the number of instruments used may have also influenced the outcomes.

## Figures and Tables

**Table 1:** Demographic and clinical features of the patients

**Variables**	**24 hours**	**48 hours**	**72 hours**	**7^th^ day**	P value*
GROUP-1	4.14±1.02	2.19±0.95	1.56±0.61	1.17±0.38	<0.001
GROUP-2	4.17±0.91	2.08±0.87	1.47±0.56	1.19±0.4	<0.001
GROUP-3	4.11±0.82	2.25±0.87	1.56±0.61	1.17±0.38	<0.001
GROUP-4	4.17±0.91	2.17±0.88	1.58±0.65	1.17±0.38	<0.001
GROUP-5	4.17±0.77	2.08±0.84	1.61±0.6	1.19±0.4	<0.001
P value	0.74 NS**	0.91 NS**	0.86 NS**	0.96 NS**	

**Table 2:** Showing comparison of Numeric Rating Scale (NRS) scores between the time periods in 5 Study Groups

**Variables**	**ProTaper Gold Group 1**	**Hyflex EDM Group 2**	**NeoEndo Flex Group 3**	**Reciproc Blue Group 4**	**WaveOne Gold Group 5**	**P value**
Age(in years)	36.67±7.27	38.25±9.43	36.36±8.9	37.06±9.39	38.17±8.11	0.836
Gender						
Male	16(44.4%)	15(41.7%)	18(50.0%)	15(41.7%)	14(38.9%)	0.441
Female	20 (55.6%)	21(58.3%)	18(50.0%)	21(58.3%)	22(61.1%)	0.523
Tooth localization						
Maxillary	18(50.0%)	16(44.4%)	16(44.4%)	14(38.9%)	17(47.2%)	0.602
Anterior	18	16	16	14	17	0.477
Posterior						
Mandibular	18(50.0%)	20(55.6%)	20(55.6%)	22(61.1%)	19(52.8%)	0.463
Anterior	9	8	7	10	8	0.619
Posterior	9	12	13	12	11	0.594

**Table 3:** Mean Number (No:) of analgesics intake between the time periods in 5(Five) Study Groups

**Variables**	**Mean No. of tablets intake**			**P value**
	**24 hours**	**48 hours**	**72 hours**	
GROUP-1	1.75±1.32	1.31±1.31	0.44±0.84	<0.01
GROUP-2	1.86±1.4	1.11±1.28	0.42±0.87	<0.001
GROUP-3	1.75±1.25	1.03±1.23	0.25±0.73	<0.002
GROUP-4	1.56±1.32	0.75±1.11	0.25±0.73	0.012
GROUP-5	1.25±1.23	0.64±0.93	0.11±0.46	0.022
P value*	0.91 NS**	0.42 NS**	0.11 NS**	
